# Lipopolysaccharide Adsorbed to the Bio-Corona of TiO_2_ Nanoparticles Powerfully Activates Selected Pro-inflammatory Transduction Pathways

**DOI:** 10.3389/fimmu.2017.00866

**Published:** 2017-08-03

**Authors:** Massimiliano G. Bianchi, Manfredi Allegri, Martina Chiu, Anna L. Costa, Magda Blosi, Simona Ortelli, Ovidio Bussolati, Enrico Bergamaschi

**Affiliations:** ^1^Department of Medicine and Surgery, University of Parma, Parma, Italy; ^2^Institute of Science and Technology for Ceramics (CNR-ISTEC), National Research Council of Italy, Faenza, Ravenna, Italy; ^3^Department of Public Health Science and Pediatrics, University of Turin, Turin, Italy

**Keywords:** lipopolysaccharide, endotoxin, titanium dioxide nanoparticles, bio-corona, macrophages, inflammation

## Abstract

It is known that the adsorption of bioactive molecules provides engineered nanoparticles (NPs) with novel biological activities. However, the biological effects of the adsorbed molecules may also be modified by the interaction with NP. Bacterial lipopolysaccharide (LPS), a powerful pro-inflammatory compound, is a common environmental contaminant and is present in several body compartments such as the gut. We recently observed that the co-incubation of LPS with TiO_2_ NPs markedly potentiates its pro-inflammatory effects on murine macrophages, suggesting that, when included in a NP bio-corona, LPS activity is enhanced. To distinguish the effects of adsorbed LPS from those of the free endotoxin, a pellet fraction, denominated P25/LPS, was isolated by centrifugation from a mixture of P25 TiO_2_ NP (128 µg/ml) and LPS (10 ng/ml) in the presence of fetal bovine serum. Western blot analysis of the pellet eluate indicated that the P25/LPS fraction contained, besides proteins, also LPS, pointing to the presence of LPS-doped NP. The effects of adsorbed or free LPS were then compared in Raw264.7 murine macrophages. RT-PCR was used to evaluate the induction of cytokine genes, whereas active, phosphorylated isoforms of proteins involved in signaling pathways were assessed with western blot. At a nominal LPS concentration of 40 pg/ml, P25/LPS induced the expression of both NF-κB and IRF3-dependent cytokines at levels comparable with those observed with free LPS (10 ng/ml), although with different time courses. Moreover, compared to free LPS, P25/LPS caused a more sustained phosphorylation of p38 MAPK and a more prolonged induction of STAT1-dependent genes. Cytochalasin B partially inhibited the induction of *Tnfa* by P25/LPS, but not by free LPS, and suppressed the induction of IRF3-dependent genes by either P25/LPS or free LPS. These data suggest that, when included in the bio-corona of TiO_2_ NP, LPS exhibits enhanced and time-shifted pro-inflammatory effects. Thus, in assessing the hazard of NP in real life, the enhanced effects of adsorbed bioactive molecules should be taken into account.

## Introduction

When introduced in organic fluids, engineered nanoparticles (NP), due to their high ratio surface/volume, adsorb proteins, lipids, and other bioactive molecules present in the medium, forming a corona that is of fundamental relevance for the interactions with cells and tissues (1–3). The biological effects of nanomaterials are markedly influenced by the bio-corona and, therefore, are expected to change in media or organic fluids of different composition (4). Conversely, the interaction with the nanomaterial may also change the conformation and/or the bioavailability of the adsorbed molecules (5), leading to enhancement or inhibition of their effects.

Although the formation and biological effects of protein corona have been extensively studied, other, non-protein bioactive molecules are expected to be adsorbed by nanomaterials. Bacterial lipopolysaccharide (LPS, endotoxin) is one of the most abundant bioactive molecules present in the environment as well as in several body compartments, such as the gut lumen. Although several mechanisms exist to limit the mucosal penetration of LPS, low endotoxin levels are also present in normal human plasma (6) and increase in several conditions (7, 8). LPS is an heat-stable component of the outer membrane of Gram-negative bacteria and works as a pathogen-associated molecular pattern, activating macrophages and promoting the production of a variety of pro-inflammatory proteins, such as tumor necrosis factor-α and other cytokines, or non-protein mediators, such as nitric oxide (NO) (9). In mammals, LPS mostly acts through transcriptional mechanisms, mediated by several, partially cross-linked transduction pathways, the most studied of which are those dependent by NF-κB and TRIF, elicited by LPS binding to the toll-like receptor 4 (TLR4). Signal transduction starts at the plasma membrane and later involves an endosomal compartment after the internalization of the complex LPS–TLR4 (10–12).

TiO_2_ NPs are considered relatively safe materials and are widely used in a variety of applications. Similar to several other types of nanomaterials (13–17), also TiO_2_ NPs have been described to adsorb LPS (18), although the amount of endotoxin adsorbed was not easily quantifiable due to interference of the nanomaterial with the assay method. Interestingly, LPS adsorption to NP has been considered one of the possible factors that interfere with cell-based immunological tests employing NP (19), because either contamination may be easily overlooked or adsorption may modulate the effects of LPS on innate immune cells (19). Investigating this latter possibility, we have recently demonstrated that the co-exposure of murine macrophages to TiO_2_ NP and LPS in protein-rich medium powerfully synergizes the pro-inflammatory effects of the endotoxin (20). The synergy was hindered by the cytoskeletal drug cytochalasin B, which inhibits endocytosis and NP internalization, and blocked by the TLR4 inhibitors polymyxin B and CLI-095. On the basis of those results, we proposed that TiO_2_ NPs adsorb LPS and enhance macrophage activation by the endotoxin *via* a TLR4-dependent mechanism that is mainly triggered from an intracellular site (20).

However, the simultaneous cell treatment with NP and LPS does not allow to understand if the fraction of LPS adsorbed to NP has different biological effects compared with free LPS, since both forms of the endotoxin are actually co-administered to the test system. In particular, it would be important to determine if free and adsorbed LPS trigger different pathways, exhibit different potencies, or change the time-course of the activation process. In order to address these questions, we have adopted here an approach based on the separation of the two fractions through centrifugation. The results indicate that the adsorption to TiO_2_ NP strongly potentiates the effects of LPS on selected transduction pathways and changes the time-course of the macrophage activation process.

## Materials and Methods

### Reagents

Fetal bovine serum (FBS) and culture media were purchased from Euro-Clone SpA (Pero, Milan, Italy). LPS from *E. coli* O55:B5 serotype and cytochalasin B were from Sigma-Aldrich (Milan, Italy) as well as all of other chemicals used in this study, whenever not specified otherwise.

### TiO_2_ NP Description, Dispersion, and Characterization

The NPs used were the TiO_2_ NP Aeroxide^®^ P25 (anatase/rutile 83/17, Evonik Industries, Degussa GmbH, Germany), produced through the flame hydrolysis Aerosil^®^ process. The physicochemical characterization of P25 under dry conditions is provided elsewhere (20). In particular, P25 have a specific surface area of 60 m^2^/g and an average crystallite size of 24 nm. TEM images of the same batch of Aeroxide^®^ P25 used in this contribution have been recently published (21).

TiO_2_ NP powder, previously heated at 230°C for 3 h for LPS decontamination, was suspended in culture medium without FBS to obtain 100× stock suspensions (12.8 mg/ml). For cell treatments, after vortexing for 30 s and a further incubation of 10 min in a Branson bath sonicator, the TiO_2_ NP stock suspension was 100-fold diluted in complete culture medium supplemented with 10% FBS so as to reach the working concentration of 128 µg/ml of NP.

**Figure 1:**
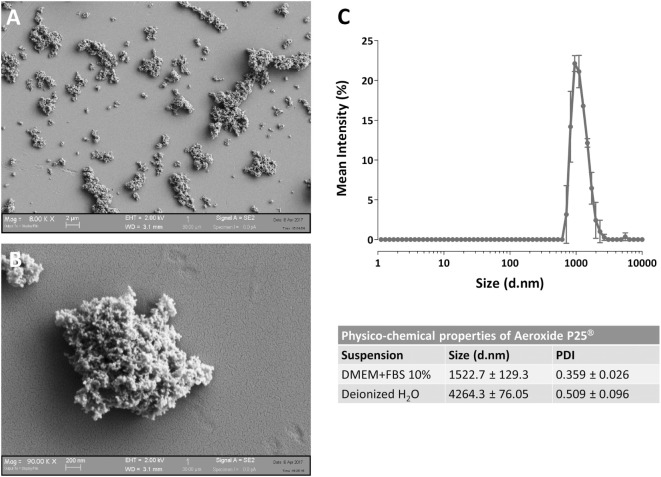
Characterization of Aeroxide^®^ P25 TiO_2_ nanoparticles (NP). **(A,B)** SEM images of TiO_2_ P25 NP dispersed in complete growth medium [Dulbecco’s modified Eagle’s medium (DMEM) + 10% fetal bovine serum (FBS)]. **(C)** Mean size distribution by intensity for P25 TiO_2_ NP (128 µg/ml) dispersed in deionized water or complete culture medium.

The determination of NP size was performed, with minor modifications, as described in Bianchi et al. (20) at the same concentrations used for the cellular tests. Particle size distribution was evaluated by dynamic light scattering (DLS) technique assessing the hydrodynamic diameter of the dispersed NPs, using ZetasizerNano ZS (Malvern Instruments, UK) with standard polystyrene cuvettes. For the evaluation of particle size, data were recorded at 25 ± 1°C, in a backscattering detection mode (scattering angle of 173°). Each result corresponds to the average of three consecutive measurements, and each measurement is the average of 15 analyses. DLS analysis provides also a polydispersity index (PDI), which is a number ranging from 0 to 1 useful to quantify the colloidal dispersion degree: samples with PDI close to 0 are considered monodispersed. The results are presented in Figure 1 and indicate that, as expected, P25 NP aggregate when suspended.

### Formation of a LPS Corona on TiO_2_ NP

**Figure 2:**
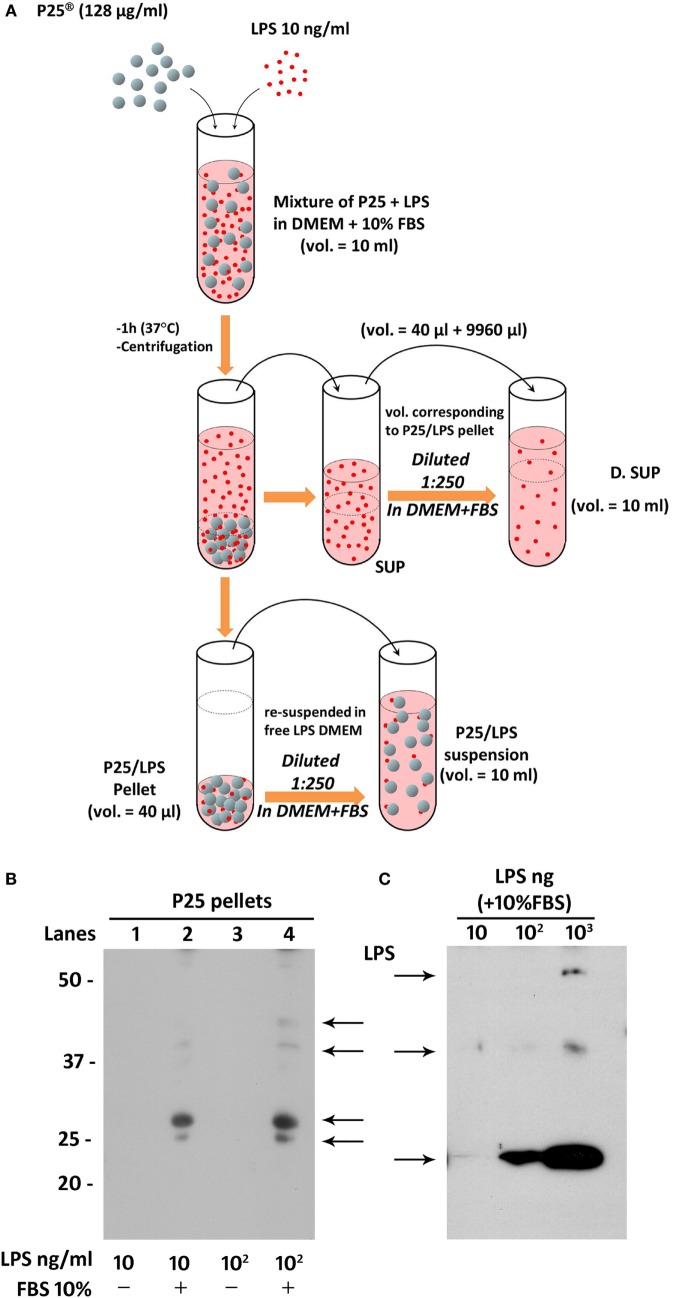
Preparation of P25/lipopolysaccharide (LPS) and free LPS fractions from a mixture of P25 TiO_2_ NP and LPS. **(A)** The cartoon provides a schematic overview of the experimental approach adopted to obtain LPS-doped P25 nanoparticle (NP). The three fractions (SUP, D. SUP, and P25/LPS suspension) result from the centrifugation of the mixture of LPS (10 ng/ml) + P25 NP (128 µg/ml) in Dulbecco’s modified Eagle’s medium (DMEM) + 10% fetal bovine serum (FBS). The P25/LPS suspension is obtained re-suspending 1:250 the P25/LPS pellet in DMEM + 10% FBS so as to obtain the same nominal dose of P25 NP present in the original mixture. For comparison, also the D.SUP has been diluted 1:250. **(B)** Detection of LPS in extracts from pellets of P25 NP (128 µg/ml) and LPS mixtures. Lanes represent the run of eluates of the following pellets (see Materials and Methods): P25 in DMEM + LPS (10 ng/ml); P25 in DMEM (+10% FBS) + LPS (10 ng/ml); P25 in DMEM + LPS (100 ng/ml); and P25 in DMEM (+10% FBS) + LPS (100 ng/ml). The blot has been performed twice with comparable results. **(C)** Detection of LPS in western blot. The indicated amounts of LPS, dissolved in Laemmli buffer 4× supplemented with 10% FBS, were treated as described under “Materials and Methods.”

Lipopolysaccharide corona formation on TiO_2_ NP (see Figure 2A) was obtained as described previously for silica NP (17) with some modifications. An aliquot of the suspension of TiO_2_ NP (128 µg/ml) was supplemented with 10 ng/ml of LPS (from a 100× stock solution in FBS-free medium) and incubated in an hybridizing oven for 1 h at 37°C under continuous rotation. The suspension was then centrifuged for 30 min at 1,900 × *g*, and the supernatant, corresponding roughly to 996/1,000 of the original volume, was transferred in a new tube and named “SUP” (for supernatant). The pellet of P25 NP (4/1,000 of the original volume) was re-suspended in complete medium at a 1:250 dilution, to attain the original working concentration of 128 µg/ml, transferred into a new tube, vortexed for 1 min, and used in the experiments as “P25/LPS” suspension. To obtain the diluted SUP (D.SUP) fraction, 40 µl of the SUP fraction was diluted 1:250 with complete medium, so as to restore the original nominal LPS concentration. Another aliquot of the suspension of TiO_2_ NP (128 µg/ml) underwent the same treatment (incubation, centrifugation, resuspension) without LPS supplementation and was used as P25 in the biological experiments.

### Western Blot of LPS in Pellet Eluates

To assess if LPS was adsorbed to P25 NP, we performed a western blot analysis against the endotoxin on the bio-corona obtained by incubating the NP with LPS. Moreover, to investigate the effect of serum proteins on LPS adsorption to NP, NP suspension and incubation with LPS was performed in the presence or in the absence of FBS 10%. Briefly, 10-ml suspensions of P25 in Dulbecco’s modified Eagle’s medium (DMEM) or in DMEM + 10% FBS were supplemented with 10 or 100 ng/ml of LPS. After centrifugation at 1,900 × *g* for 30 min, pellets were eluted in 40 µl of Laemmli buffer 1× (62.5 mM Tris–HCl, pH 6.8, 2% SDS, 10% glycerol, and 0.1 M DTT) and transferred in a clean Eppendorf tube. After heating at 95°C for 10 min, tubes were centrifuged at 12,000 rpm for 5 min. The combined eluates of two pellets (approximately 80 µl) were loaded on a 12% gel for SDS-PAGE. After the run, separated components were transferred to PVDF membranes (Immobilon-P, Millipore, Millipore Merck Corporation, MA, USA). Non-specific binding sites were blocked with an incubation of 1 h at room temperature in blocking solution (Western Blocking Reagent, Roche) diluted in Tris buffered saline (TBS, pH 7.5). The blots were then exposed at 4°C overnight to goat anti-LPS polyclonal antibody (Abcam, Cambridge, UK) diluted in the blocking solution at 1:500. After washing, the blots were exposed for 1 h at room temperature to HRP-conjugated anti-goat antibody (Cell Signaling), diluted 1:20,000 in blocking solution. Immunoreactivity was visualized with Westar HRP Substrate (Cyanagen srl, Bologna, Italy).

The results, reported in Figure 2B, showed an increase of LPS-positive bands for the samples with 10 (lane 2) and 100 ng/ml LPS (lane 4) at several apparent molecular weights. No bands were instead detectable for the eluates from NP incubated with the same doses of LPS but in the absence of FBS 10% (lanes 1 and 3). In parallel, the specificity of the antibody was validated in Figure 2C, through the western blot of different doses of LPS dissolved in Laemmli buffer 4×, supplemented with 10% FBS.

### Cell Cultures and Experimental Treatments

Murine peritoneal macrophages Raw264.7 from the Istituto Zooprofilattico della Lombardia e dell’Emilia Romagna (Brescia, Italy) were cultured in DMEM completed with FBS 10%, streptomycin (100 µg/ml) and penicillin (100 U/ml) and 4 mM of glutamine. Cells were routinely cultured in 10-cm dishes under humidified atmosphere in the presence of 5% CO_2_ in air. For the experiments, cells were seeded in 24-well plates at a density of 75 × 10^3^/cm^2^ (20). After 24 h in culture, Raw264.7 cells were exposed to 128 µg/ml (corresponding to 80 µg/cm^2^ of culture surface) of P25 NP, in the presence or in the absence of 10 ng/ml of LPS, to LPS (10 ng/ml), or to 128 µg/ml of P25/LPS NP for the times indicated in each experiments. Under these conditions, cell viability was not significantly affected by the experimental treatments (20).

### Nitrite Determination

The determination of nitrite concentration in culture medium was performed following the method described in Ref. (22). After 24 h of exposure of Raw264.7 to the experimental treatment, 100 µl of culture medium was transferred to black 96-well plates with a clear bottom (Corning, Cambridge, MA, USA). 20 µl of a solution of 0.025 mg/ml in 0.31 M HCl of 2,3-diaminonaphthalene (Invitrogen, Life Technologies, Monza, Italy) were then added and, after 10 min at room temperature, the reaction was stopped with 20 µl of 0.7 M NaOH. Standards were performed in the same medium from a solution of 1 mM sodium nitrite. Fluorescence was determined with a multimode plate reader Perkin Elmer Enspire.

### Western Blot of Proteins Involved in Transduction Pathways

Total protein extracts were obtained as previously described (20). Briefly, macrophages were homogenized in 70 µl of lysis buffer (20 mM Tris–HCl, pH 7.5, 150 mM NaCl, 1 mM EDTA, 1 mM EGTA, 1% Triton, 2.5 mM sodium pyrophosphate, 1 mM β-glycerophosphate, 1 mM Na_3_VO_4_, 1 mM NaF, 2 mM imidazole) supplemented with a protease inhibitors cocktail (Complete, Mini, EDTA-free, Roche, Monza, Italy). Lysates were transferred in Eppendorf tubes and mixed with 23 µl (1/3 of the lysate total volume) of Laemmli buffer 4× (250 mM Tris–HCl, pH 6.8, 8% SDS, 40% glycerol, and 0.4 M DTT). After heating at 95°C for 10 min, 30 µl of each samples was loaded on a 10% gel for SDS-PAGE. Separated proteins were transferred to PVDF membranes (Immobilon-P, Millipore, Millipore Merck Corporation, MA, USA). Non-specific binding sites were blocked with an incubation of 1 h at room temperature in blocking solution (Western Blocking Reagent, Roche) diluted in TBS (pH 7.5). The blots were then exposed at 4°C overnight to the following antibodies diluted in 5% BSA in TBST (Tween 20 0.1% in TBS): anti-Nos2 (rabbit polyclonal, 1:400, Santa Cruz Biotechnology, Santa Cruz, CA, USA); anti-p-p38 (rabbit polyclonal, 1:500, R&D Systems, Minneapolis, MN, USA); anti-p38 (rabbit polyclonal, 1:500, R&D Systems); anti-p-ERK (rabbit polyclonal, 1:500, R&D Systems); anti-ERK (rabbit polyclonal, 1:500, R&D Systems); anti-p-IRF3 (rabbit polyclonal, 1:500, Biorbyt, Cowley Road, Cambridge, UK); anti-IRF3 (rabbit polyclonal, 1:1,000, Cell Signaling Technology, Danvers, MA, USA); anti-p-STAT1 (rabbit polyclonal, 1:1,000, Cell Signaling); anti-STAT1 (rabbit polyclonal, 1:1,000, Cell Signaling); anti-p-c-JUN (rabbit polyclonal, 1:1,000, Millipore Merck); anti-c-JUN (rabbit antiserum, 1:1,000, Millipore Merck); and anti-beta actin (mouse monoclonal, 1:2000, Santa Cruz Biotechnology). After washing, the blots were exposed for 1 h at room temperature to HRP-conjugated anti-rabbit or anti-mouse antibodies (Cell Signaling), diluted 1:20,000 in blocking solution. Immunoreactivity was visualized with Immobilon Western Chemiluminescent HRP Substrate (Millipore, Merck).

### RT-PCR

Total RNA was isolated with GenElute Mammalian Total RNA Miniprep Kit (Sigma-Aldrich) as described in Ref. (20). After reverse transcription, aliquots of cDNA from each sample were amplified in a total volume of 25 µl with the Go Taq PCR Master Mix (Promega Italia, Milan, Italy), along with the forward and reverse primers (5 pmol each) reported in Table 1. Real-time PCR was performed in a 36-well RotorGeneTM3000, version 5.0.60 (Corbett Research, Mortlake, VIC, Australia). For all the messengers to be quantified, each cycle consisted of a denaturation step at 95°C for 20 s, followed by separate annealing (30 s) and extension (30 s) steps at a temperature characteristic for each pair of primers (Table 1). Fluorescence was monitored at the end of each extension step. Melting curve analysis was added at the end of each amplification cycle. Data analysis was made according to the relative standard curve method. Expression data were reported as the ratio between each investigated mRNA and *Gapdh* mRNA.

**Table 1:** Primers and temperatures of annealing adopted for RT-PCR experiments.

Gene	Forward	Reverse	T (°C)	Amplicon size (bp)
Tumor necrosis factor alpha *(Tnfa)*	5′-CCCTCACACTCAGATCATCTTCT-3′	5′-GCTACGACGTGGGCTACAG-3′	55°C	61
Interferon beta 1 *(Ifnb)*	5′-CAGCTCCAAGAAAGGACGAAC-3′	5′-GGCAGTGTAACTCTTCTGCAT-3′	56°C	138
Interferon-induced protein with tetratricopeptide repeats 2 *(Ifit2)*	5′-AGAACCAAAACGAGAGAGTGAAG-3′	5′-TCCAGACGGTAGTTCGCAATG-3′	57°C	106
Glyceraldehyde 3-phosphate dehydrogenase *(Gapdh)*	5′-TGT TCC TAC CCC CAA TGT GT-3′	5′-GGT CCT CAG TGT AGC CCA AG-3′	57°C	137

### Confocal Microscopy of Live Cells

Raw264.7 cells were seeded on four-well chambered coverglasses at a density of 75 × 10^3^/cm^2^. The day after, cells were incubated in the presence or in the absence of cytochalasin B (20 µM, Sigma-Aldrich) for 1 h and then stained with LysoTracker™ Red DND-99 (70 nM, Molecular Probes, Life Technologies) and calcein-AM (1 µM, Millipore Merck) for 2 h. Stained cells were then treated with 5 µg/cm^2^ of P25 NP or P25/LPS and imaged by an inverted LSM 510 Meta (Carl Zeiss, Jena, Germany) while maintained at 37°C, 5% CO_2_ in a Kit Cell Observer (Carl Zeiss, Jena, Germany) (23–25). Single-plane confocal images were taken at 24 h of treatment using a 40× (1.3 NA) oil objective. Excitation at 633 nm and reflectance were used to visualize P25 TiO_2_ NPs; excitation at 543 nm and emission recorded through a 580- to 630-nm band pass barrier filter were used for LysoTracker™ to visualize lysosomes; excitation at 488 nm and emission through a 515- to 540-nm band pass filter were used for calcein to visualize cytoplasm.

### Statistical Analysis

Data are expressed as means ± SD. For nitrite determination experiments, the statistical analysis was performed through one-way ANOVA for multiple comparisons, applying the Bonferroni correction. In all the other experiments, a two-tail Student’s *t*-test for unpaired data was adopted. Graph Pad Prism™ software version 6.00 (Graph Pad Software Inc., San Diego, CA, USA) was used. Results were considered significant with *p* < 0.05.

## Results

### P25/LPS Strongly Potentiates the LPS-Dependent NO Production and Nos2 Protein Expression in Murine Macrophages

**Figure 3:**
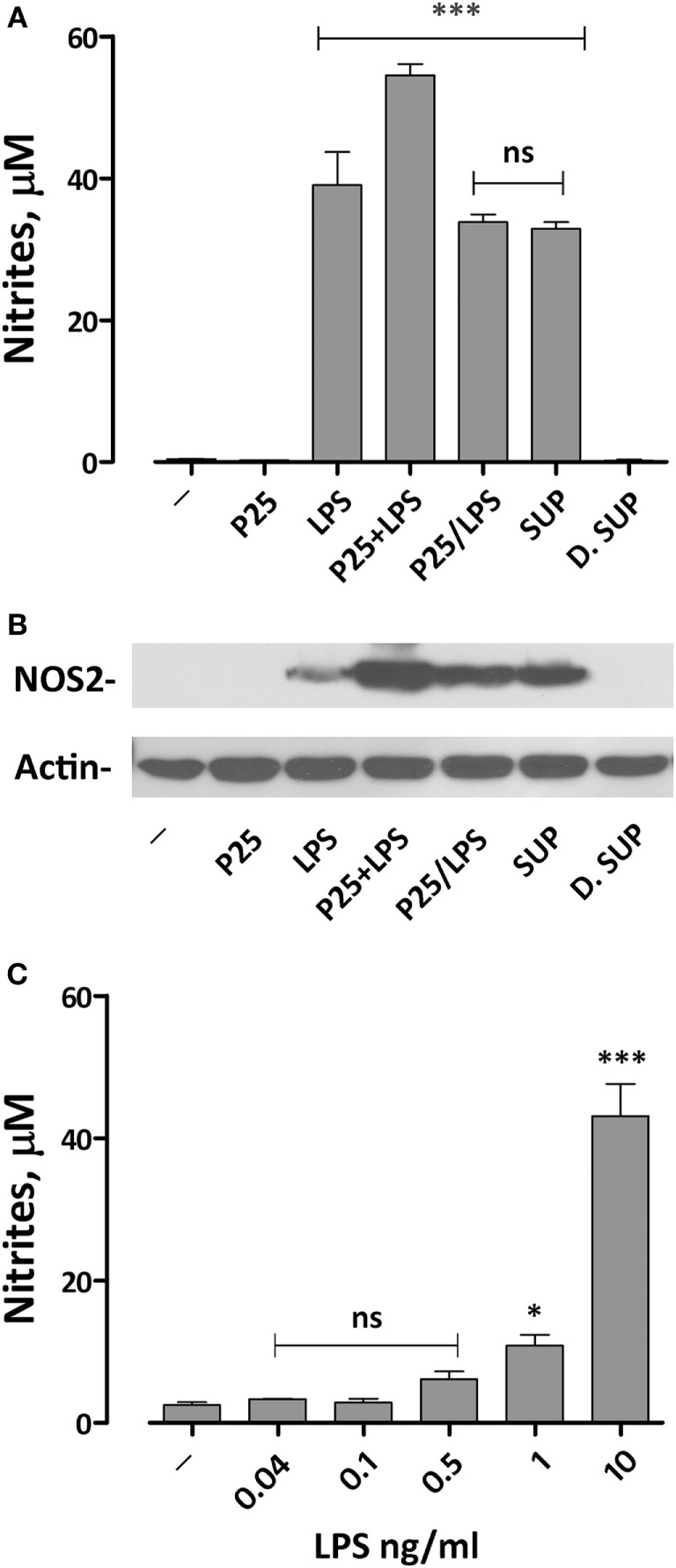
Effects of the exposure to P25/lipopolysaccharide (LPS) on nitric oxide production and Nos2 expression in macrophages. Raw264.7 cells were incubated for 24 h in Dulbecco’s modified Eagle’s medium (DMEM) + 10% fetal bovine serum (FBS) in the presence of P25 (128 µg/ml), LPS (10 ng/ml), the mixture of P25 and LPS (128 µg/ml + 10 ng/ml, respectively), P25/LPS (the pellet of the spun mixture, re-suspended at the original volume, with nominal doses of 128 µg/ml, for TiO_2_ NP, and 40 pg/ml for LPS), the undiluted (SUP, nominal LPS dose 10 ng/ml), or diluted (D.SUP, nominal LPS dose of 40 pg/ml) supernatant of the spun mixture. **(A)** At the end of the incubation, the culture medium was harvested to determine nitrite concentration. **(B)** The same cell monolayers were lysed to evaluate Nos2 expression through WB analysis. The experiment has been performed twice with comparable results. **(C)** Raw264.7 cells were incubated for 24 h in the presence of the indicated doses of LPS in DMEM + 10% FBS. At the end of the incubation, the culture medium was harvested to determine nitrite concentration. For **(A,C)**, data are means of four independent determinations ± SD. For **(A)**, ***, *p* < 0.001 vs. cells treated with P25; ns, not significant vs. LPS alone, as evaluated by one-way ANOVA for multiple comparisons with Bonferroni correction. For **(C)**, *, ***, *p* < 0.05, *p* < 0.001 vs. LPS-untreated cells; ns, not significant vs. LPS-untreated cells, as evaluated by one-way ANOVA for multiple comparisons with Bonferroni correction.

We have previously demonstrated that the simultaneous exposure to TiO_2_ NP and LPS synergized the pro-inflammatory effects of the two compounds (20). To assess if we could reproduce this effect with the P25/LPS fraction, nitrite medium concentration (as a proxy of NO production) and Nos2 expression were evaluated after 24 h of exposure of Raw264.7 cells to TiO_2_ NP, LPS, the mixture of NP, and LPS, and the two fractions resulting from the centrifugation of the mixture (the re-suspended pellet and the supernatant, see Figure 2A for details). The results, presented in Figure 3A, indicate that, while P25 NP alone did not significantly affect NO production, the P25/LPS fraction (nominal concentration of LPS 40 pg/ml) caused a huge increase in nitrite concentration in culture medium. The TLR4-dependence of the effect was confirmed by its suppression in cells treated with polymyxin B (50 µg/ml, results not shown). The magnitude of the effect was not significantly different to that caused by LPS alone, while it was smaller than that observed upon a simultaneous incubation with LPS and P25 NP. Free LPS in the undiluted supernatant fraction (nominal concentration of 10 ng/ml) also caused a comparable increase in nitrites. On the contrary, when diluted at the same ratio used for P25/LPS (1:250, nominal concentration of LPS 40 pg/ml), the supernatant fraction did not stimulate macrophages to produce NO. Consistently, results presented in Figure 3C indicate that free LPS at 40 pg/ml did not increase NO production that required a dose of LPS of at least 1 ng/ml.

Functional data were consistent with the results obtained by western blot. Indeed, Nos2 protein expression was markedly increased in cells exposed to P25/LPS, at levels comparable with those observed with LPS alone and only slightly lower than those observed after exposure to the mixture LPS plus NP (Figure 3B). As expected, the diluted supernatant (free LPS) did not affect the expression of Nos2.

### P25/LPS Promotes the Activation of both NF-κB/AP1 and IRF3-Dependent Genes

**Figure 4:**
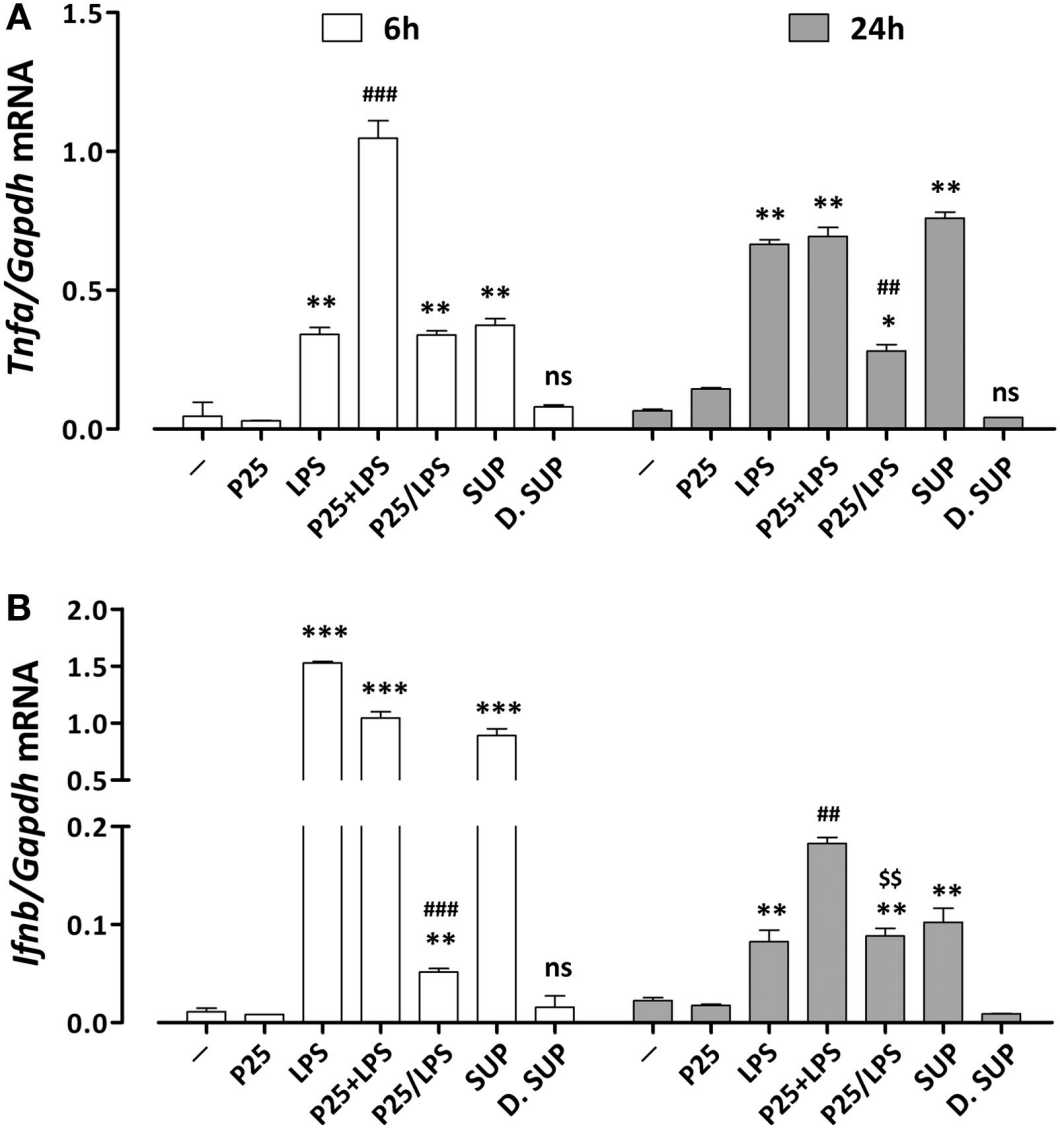
Assessment of the pro-inflammatory gene response elicited by P25/lipopolysaccharide (LPS) in macrophages. mRNA expression levels of *Tnfa* **(A)** and *Ifnb* **(B)** were assessed in Raw264.7 cells after 6 and 24 h of exposure to the indicated experimental conditions (see legend to Figure 2 for details). Control cells (−) were incubated in plain serum-supplemented medium. Data are means of two (for *Tnfa)* or three (for *Ifnb*) independent experiments each performed in duplicate with SD shown. *, **, *** *p* < 0.05, *p* < 0.01, *p* < 0.001 vs. cells treated with P25 at the same experimental time; ^##^, ^###^, *p* < 0.01, *p* < 0.001 vs. cells treated with LPS at the same experimental time; ^$$^, *p* < 0.01 vs. cells treated with P25/LPS at 6 h, as evaluated by two-tailed *t*-test for unpaired data.

The induction of pro-inflammatory genes is the final result of a cascade of intracellular signals that involves different transduction pathways. To assess the effect of P25/LPS on the induction of pro-inflammatory genes dependent on different pathways, the expression of *Tnf*α and *Ifnb* was evaluated after 6 and 24 h of treatment by RT-PCR. While *Tnf*α expression is controlled by the MyD88–NF-κB–AP1-dependent pathway (26), *Ifnb* gene is mainly dependent on the TRIF pathway through the activation of IRF transcription factors (27). As reported in Figure 4A, the mixture of P25 and LPS markedly potentiated the LPS-dependent *Tnfa* induction after 6 h of exposure. While P25 NP alone did not affect significantly gene expression, the P25/LPS fraction induced *Tnfa* at levels comparable to those stimulated by free LPS. A comparable induction of *Tnfa* was also produced by the undiluted supernatant fraction, while the diluted supernatant was without effect. At 24 h *Tnfa* expression was further increased in cells treated with free LPS and undiluted supernatant, while it decreased in cells treated with the mixture and remained fairly stable with the P25/LPS fraction. The induction of *Ifnb* (Figure 4B) was clearly detectable after 6 h of exposure to free LPS, the mixture of LPS and NP, or the undiluted supernatant, while it was much lower, although significant, in cells treated with the P25/LPS fraction. When studied after 24 h of exposure, *Ifnb* mRNA levels markedly decreased in cells treated with free LPS, the mixture of LPS and NP, or the undiluted supernatant but, conversely, increased in cells incubated with the P25/LPS fraction. Also for this gene the diluted supernatant had no stimulatory effects.

### P25/LPS and Free LPS Differentially Activate Intracellular Transduction Pathways

**Figure 5:**
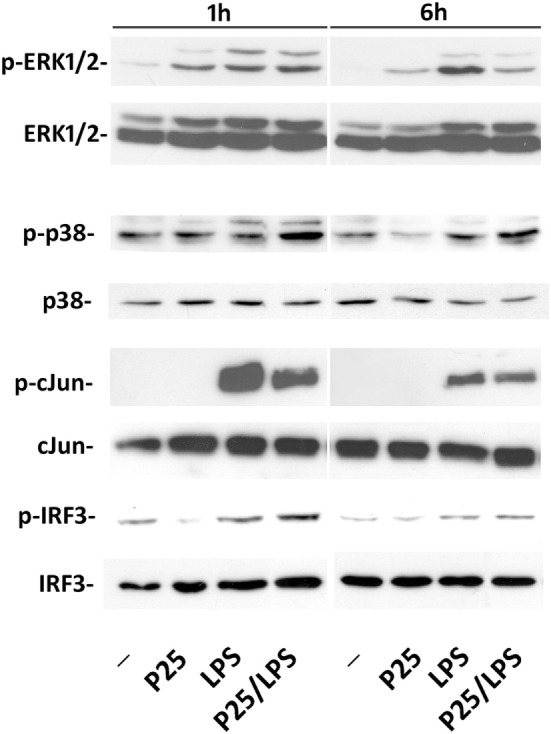
Evaluation of intracellular transduction pathways triggered by P25/LPS. Raw264.7 cells were incubated in the presence of P25 (128 µg/ml), LPS (10 ng/ml), or P25/LPS (nominal doses of 128 µg/ml, for TiO_2_ NP, and 40 pg/ml, for LPS). At the end of the incubation, the activation of ERK1/2, p38, Jun, and IRF3 was evaluated through the WB analysis of their phosphorylated forms. Representative experiments, performed twice with comparable results, are shown.

The representative experiment shown in Figure 5 reports the effect of P25/LPS and free LPS on several components of the transduction pathways activated by LPS. MAPK cascade is an integral part of the MyD88-dependent transduction pathway that ends in the phosphorylation of Jun and the subsequent activation of the transcription factor AP1. One hour after medium replacement, the ERK1/2 branch of the MAPK pathway was activated in cells incubated with P25, LPS, and P25/LPS, as revealed by the high abundance of the phosphorylated forms. ERK1/2 activation persisted at 6 h in cells incubated with LPS while decreased in cultures exposed to either P25 or P25/LPS. In contrast, P25/LPS produced a clearly larger activation of p38 than LPS at both experimental times. The JNK substrate Jun was strongly activated by LPS at 1 h of treatment and, at a lesser degree, by P25/LPS. In both conditions, Jun phosphorylated form decreased at 6 h. As expected by *Ifnb* induction, a clear-cut phosphorylation of IRF3 was detected after 1 h of treatment in cells incubated with either free LPS or P25/LPS. In both conditions, activation of IRF3 decreased at 6 h, but it was still more evident in cells exposed to P25/LPS compared to LPS-treated cells extracted at the same time.

### P25/LPS Shifts the Kinetics of LPS-Dependent *Ifit2* Induction

**Figure 6:**
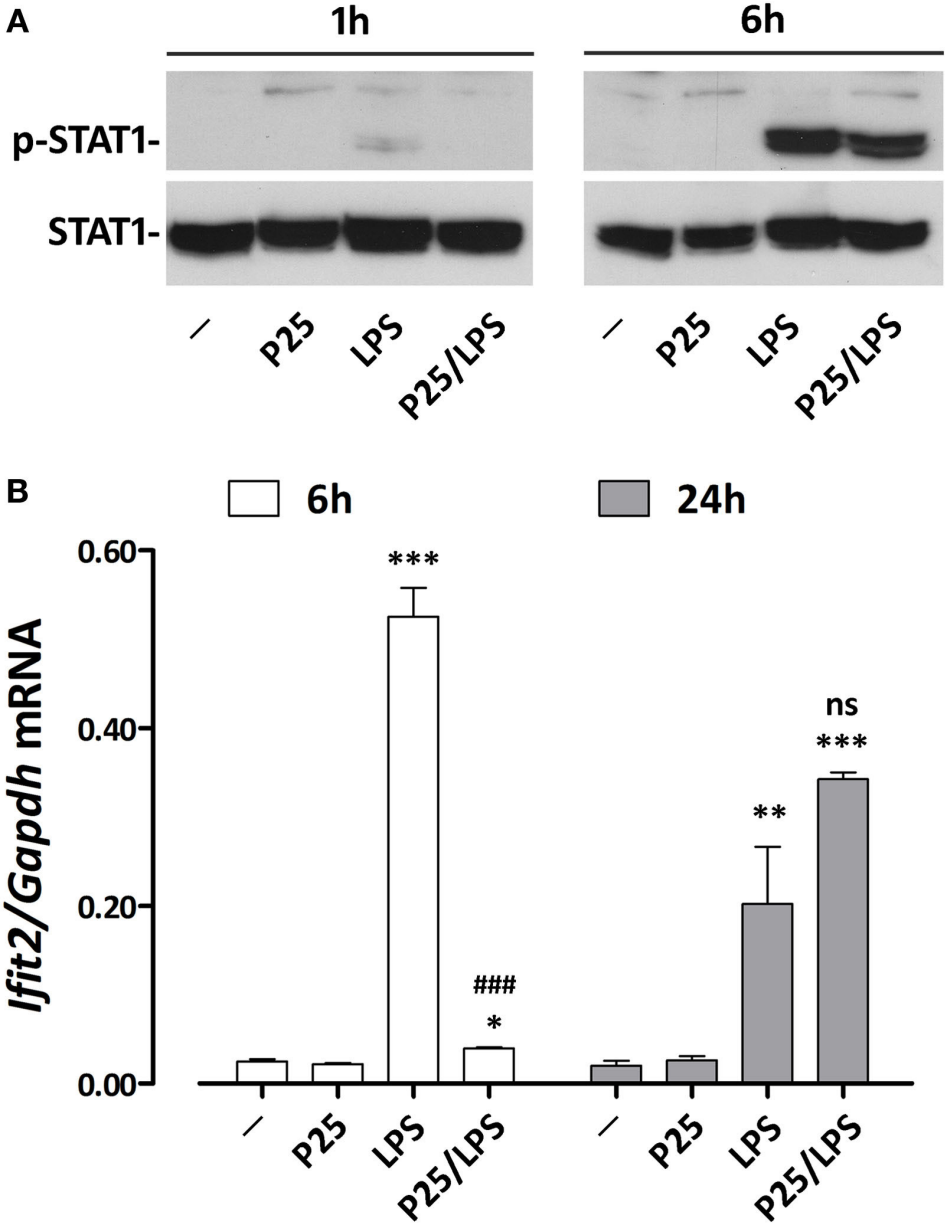
P25/LPS induces *Ifit2*. Raw264.7 cells were incubated for the indicated times in DMEM + 10% FBS supplemented with P25 (128 µg/ml), LPS (10 ng/ml), or P25/LPS (nominal doses of 128 µg/ml, for TiO_2_ NP, and 40 pg/ml, for LPS). Control cells (−) were incubated in plain DMEM + 10% FBS. **(A)** After 1 or 6 h, the activation of STAT1 was evaluated through WB analysis. A representative experiment, performed twice with comparable results, is shown. **(B)** After 6 or 24 h *Ifit2* expression was assessed by RT-PCR. Data are means of two independent experiments, each performed in duplicate, with SD shown. *, **, ***, *p* < 0.05, *p* < 0.01, or *p* < 0.001 vs. cells treated with P25 at the same experimental time; ^###^, *p* < 0.001 vs. cells treated with LPS at the same experimental time, as evaluated by two-tailed *t*-test for unpaired data.

To verify if adsorption to P25 NP effectively changes the time-course of *Ifnb* induction, we investigated the expression of *Ifit2*, an IFNβ-induced gene that encodes a member of a group of proteins responsible for the inhibition of viral replication (28). As many other IFN-dependent genes, *Ifit2* induction is activated by the phosphorylation of STAT transcription factors. STAT1 phosphorylation was already detectable after 1 h of incubation in cells treated with free LPS but not with P25/LPS. In contrast, after 6 h, a massive STAT1 activation was evident in cells treated with either LPS or P25/LPS (Figure 6A). Consistently, also *Ifit2* induction followed a different time-course in the two experimental conditions (Figure 6B). In cells incubated with free LPS, a huge increase of *Ifit2* mRNA was evident after 6 h of incubation while P25/LPS was completely ineffective at this time point. In contrast, at 24 h of treatment, P25/LPS caused a larger *Ifit2* induction than free LPS.

### Cytoskeleton Disruption and Internalization Inhibition Differentially Affect the Induction of TRIF-Dependent and -Independent Genes

**Figure 7:**
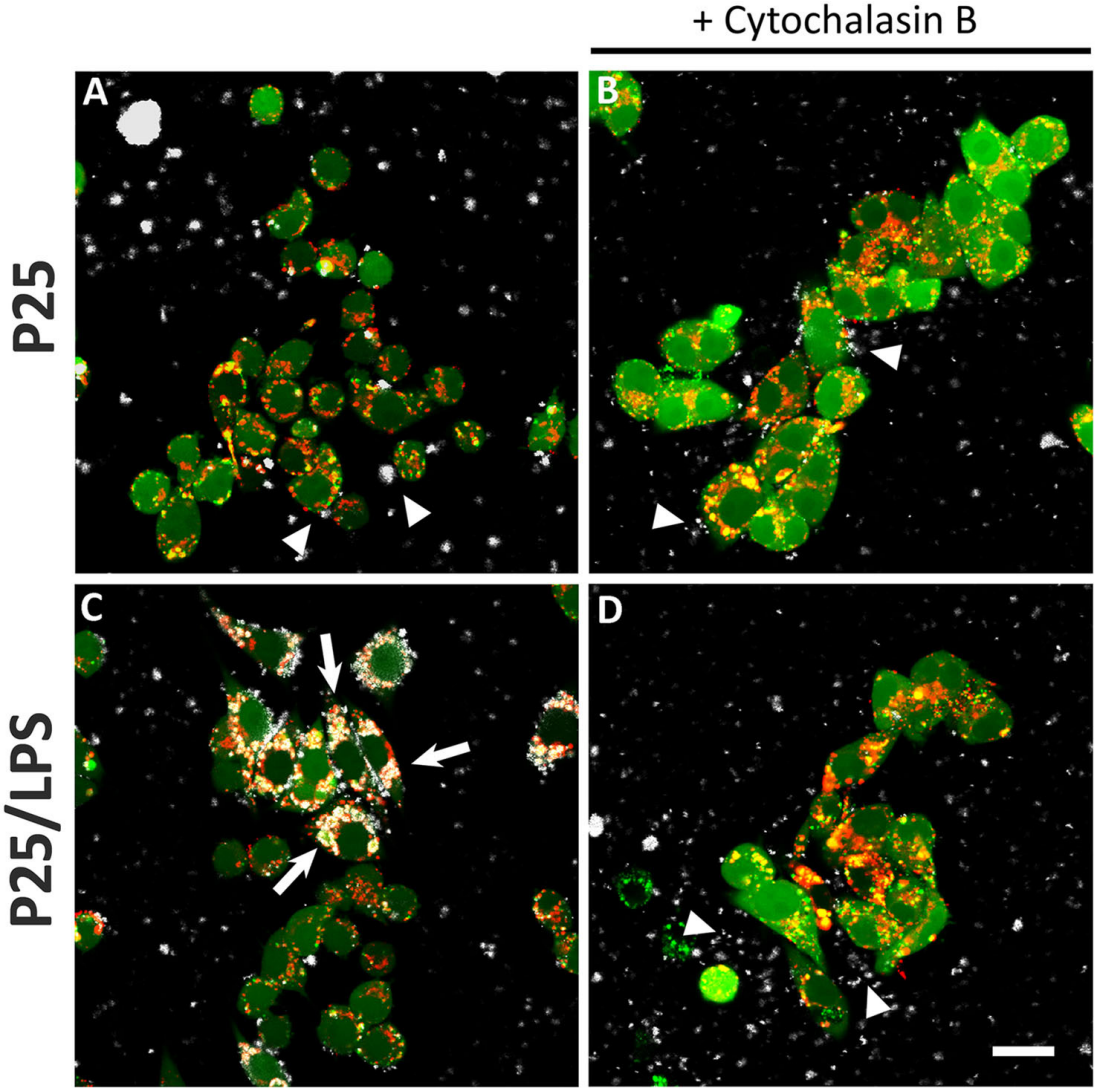
Internalization of TiO_2_ P25 and P25/LPS. Raw264.7 cells were preincubated for 1 h in the absence **(A,C)** or in the presence of cytochalasin B [20 µM **(B,D)**] and then exposed to P25 [16 µg/ml **(A,B)**] or P25/LPS [nominal dose 16 µg/ml **(C,D)**] for further 24 h. At the end of the incubation, cell monolayers were stained with calcein-AM, for cytoplasm and nucleus, and LysoTracker^®^, for lysosomes, as reported in “Materials and Methods.” For each condition, a single horizontal confocal section of a representative field is shown. P25 or P25/LPS, white; cells, green; lysosomes, red. The experiment has been performed twice with similar results. Arrows, internalized NP aggregates. Arrowheads, extracellular NP aggregates in contact with the cell membrane. Bar = 20 µm.

The confocal images (Figure 7) show the effects of LPS adsorption on TiO_2_ NP internalization. Under our experimental conditions, most of the P25 NP formed aggregates that are well evident from the reflected light (Figures 7A–D, white). P25, which were preheated at 230°C so as to eliminate LPS before the experiment, were scarcely internalized by Raw264.7 macrophages, as indicated by their prevalent visualization in the extracellular compartment (Figure 7A, arrowheads). Consistently, in the same field, lysosomes were red or yellow (indicating a partial co-localization with calcein). On the contrary, P25/LPS were massively taken up by cells and internalized in discrete compartments (Figure 7C, arrows), in some of which a co-localization with the lysosomal marker was evident. Under this condition, most of the NP in the field were intracellular. As expected, cytochalasin B blocked the internalization, although P25 NP aggregates were detected in close contact with the cells (Figure 7D, arrowheads).

**Figure 8:**
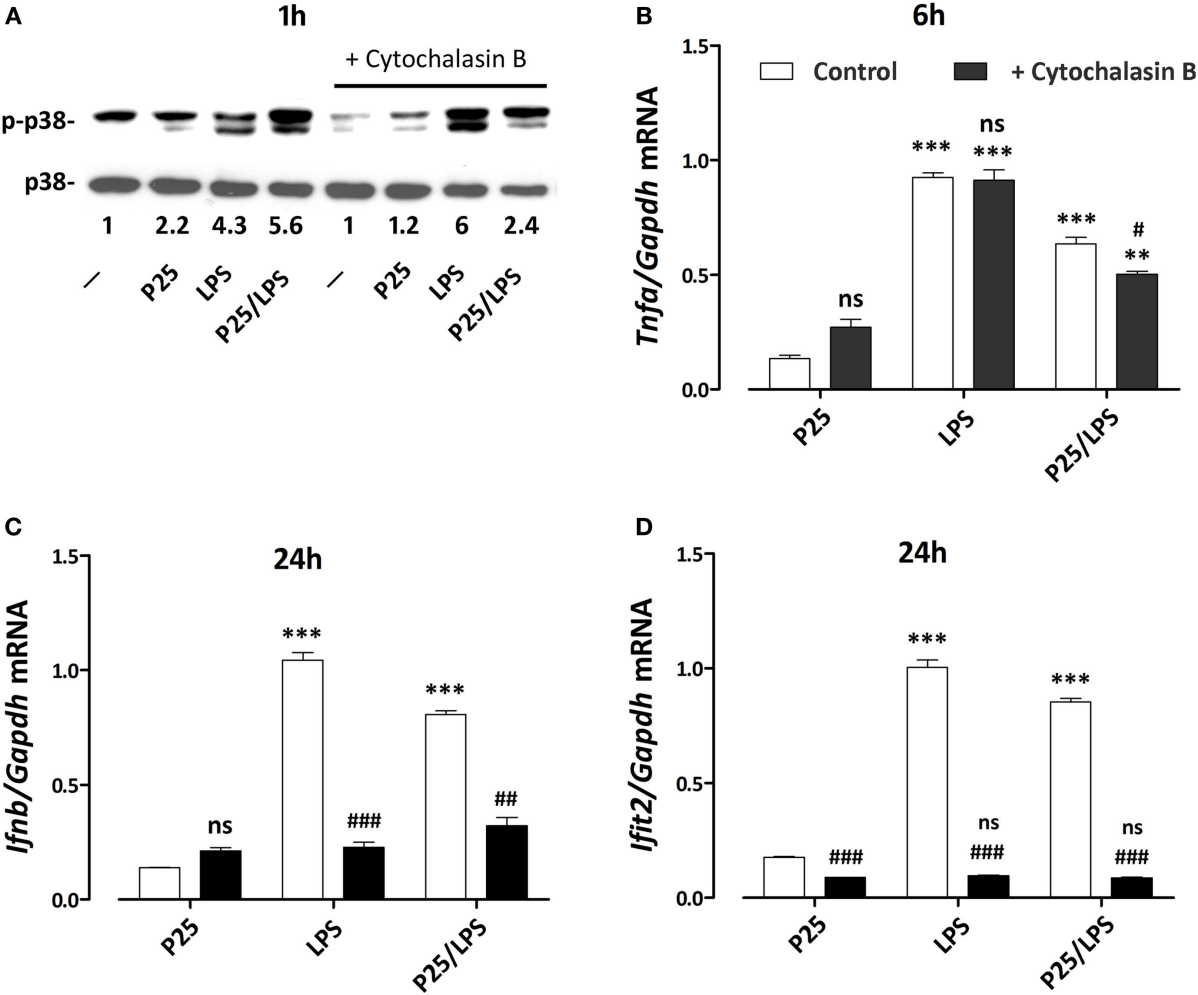
Effects of cytochalasin B on p38 phosphorylation and pro-inflammatory gene induction. Raw264.7 cells, preincubated for 1 h in the absence or in the presence of cytochalasin B (20 µM), were incubated for the indicated times in the presence of P25 (128 µg/ml), lipopolysaccharide (LPS) (10 ng/ml), or P25/LPS (nominal doses of 128 µg/ml, for TiO_2_ NP, and 40 pg/ml, for LPS). At the indicated times, the phosphorylation status of p38 **(A)** and the expression of *Tnfa* **(B)**, *Ifnb* **(C)**, and *Ifit2* **(D)** were evaluated by western blot or RT-PCR, respectively. For **(A)**, a representative experiment, performed twice with comparable results, is shown. The numbers represent the quantification of the bands ratio (p-p38/p38) with control cells kept at 1. For **(B–D)**, data are means of two independent experiments, each performed in duplicate, with SD shown. **, ***, *p* < 0.01 or *p* < 0.001 vs. corresponding cells (preincubated w/wo cytochalasin B) treated with P25; ^#^, ^##^, ^###^, *p* < 0.05, *p* < 0.01, *p* < 0.001 vs. cells under the same experimental condition without cytochalasin B, as evaluated by two-tailed *t*-test for unpaired data.

To assess if the signal triggered by LPS or P25/LPS depends on internalization, p38 activity was investigated in cells exposed to free LPS or P25/LPS with or without a 1 h-pretreatment with cytochalasin B. The results (Figure 8A) indicate that inhibition of internalization did not impair the activation of p38 by free LPS, which was even increased, but markedly hindered the P25/LPS effect on the kinase. LPS-dependent stimulation of *Tnfa* expression, assessed at 6 h of exposure (Figure 8B), was not affected at all by cytoskeletal disruption, while a small, but significant inhibition was observed in P25/LPS-treated cells. On the contrary, cytochalasin B completely prevented the induction of both *Infb* (Figure 8C) and *Ifit2* (Figure 8D) by either LPS or P25/LPS.

## Discussion

Once introduced into the body, engineered nanomaterials adsorb biomolecules from the biological fluids. Thus, the biological effects observed *in vivo* could be due not only to the synthetic identity of the materials but also to the bioactive agents adsorbed on their surface (29). As far as LPS is concerned, experimental evidence describing LPS adsorption by different types of NP has been already reported, although the biological consequences of the interaction are controversial, with enhancing or mitigating effects reported in different contributions (13–17). These conflicting observations may be related to particular physico-chemical features of the NP tested. In particular, NP endowed with a low PDI tend to float in culture medium and may act as LPS quenchers in cultures of adherent cells. On the contrary, NP endowed with a high PDI in biological media, would work as LPS deliverers, given their tendency to aggregate and precipitate on adherent cells. Under the experimental conditions adopted here, P25 NP markedly aggregate and, as visualized with the confocal images, come in close contact with the cell monolayer, Thus, the bioavailability of adsorbed LPS would be enhanced.

In agreement with these arguments, we had observed that TiO_2_ P25 NP, co-administered with LPS, strongly enhanced the pro-inflammatory response triggered in murine macrophages by the endotoxin, a result that could be attributed to the presence of LPS adsorbed to NP (20). In order to discriminate the roles played by free LPS or LPS adsorbed on P25 NP in macrophage activation, we treated Raw264.7 cells with spun P25 NP obtained from a NP suspension preincubated in complete serum-supplemented medium in the presence of LPS. The pellet of P25/LPS has been re-suspended in the original volume of LPS-free medium, and its effects on macrophages were compared with those observed after exposure to free LPS. We avoided the washing of the pellet, so as to closely mimic the situation *in vivo*, in which cells are exposed to nanomaterials suspended in high-protein media endowed with both hard and soft corona (30, 31). In the biological matrix adopted here (DMEM supplemented with 10% FBS), LPS may be adsorbed to the NP bio-corona through its binding to proteins, thus interacting only indirectly with the NP. The western blot analysis shown in Figure 2B indicates that LPS can be indeed eluted from the spun TiO_2_ NP (the P25/LPS fraction). Interestingly, the endotoxin was detectable only if the incubation of the NP and LPS had been performed in the presence of serum, suggesting that proteins actually promote the adsorption of the endotoxin to the NP bio-corona. This means that the material that interacts with the cells does not consist of a binary complex aggregated NP–LPS but, most likely, of a ternary complex aggregated NP–serum proteins–LPS. Further investigations are needed to ascertain if the presence of LPS changes the quality or quantity of the serum proteins adsorbed and how the adsorbed proteins modulate the biological effects of the endotoxin.

In the previous paper, we limited our analysis to pro-inflammatory genes the induction of which relies on the activation of MyD88-dependent transduction pathways and AP1/NF-κB-dependent transcription, such as *Tnfa* and *Nos2* (20). In this contribution, besides confirming those effects (Figures 3, 4 and 8), we have studied pro-inflammatory genes, such as *Ifnb* and *Ifit2*, mainly dependent upon the activation of the TRIF pathway and IRF transcription factors. Also for these genes, P25/LPS was highly effective (Figures 4, 6 and 8), leading to a level of gene induction comparable with that caused by 10 ng/ml of free LPS, at least at later times of incubation. We have not extended our analysis to proteins products, since, being interested in mechanisms and time-course of signal transduction, changes in gene expression at mRNA levels is the earliest output while protein levels could be influenced by other regulatory mechanisms.

The activation of IRF3 and STAT1 was also quantitatively comparable in cells treated with free LPS and P25/LPS at 6 h of treatment (Figures 5 and 6). Given the very high dilution of the P25/LPS fraction before exposure (see Figure 2A), the clear-cut effects observed cannot be attributed to the residual free LPS (the nominal concentration of which would be 40 pg/ml), since free LPS at that concentration was without effect (Figure 3C). However, these results are consistent with the presence of LPS in the NP bio-corona, provided that adsorption increases the quantity of LPS present in the P25/LPS fraction or enhances its biological effects. We were not able to quantify this fraction either with silver staining or with Limulus test (not shown), possibly for the interference of the NP with the test. However, it is likely that this aliquot is low, as suggested by the substantially comparable effects (Figures 3 and 4) of free LPS (at a concentration of 10 ng/ml) and the SUP fraction, which is the supernatant resulting from the centrifugation of the mixture NP + LPS (see Figure 4A).

Once included in the NP bio-corona, LPS caused a prolonged activation of p38 MAPK, while free LPS promoted a more evident activation, also at later times, of the ERK1/2 branch of MAPK (Figure 5). These data, consistent with the results reported by others with gold NPs (32), suggest that a differential activation of the two MAPK branches occurs if LPS is free or adsorbed to NP. Moreover, when compared to free endotoxin, P25/LPS had delayed effects on STAT1 activation and *Ifit2* induction (Figure 6), two typical IFNβ-dependent effects (33). Indeed, *Ifnb* induction was slower, but more stable, in cells treated with P25/LPS compared to free LPS (Figure 4). Conversely, as far as *Tnfa* is concerned, free LPS triggered a more prolonged gene induction. Taken together, these results highlight a different time-course of effects of P25/LPS and free LPS.

**Figure 9:**
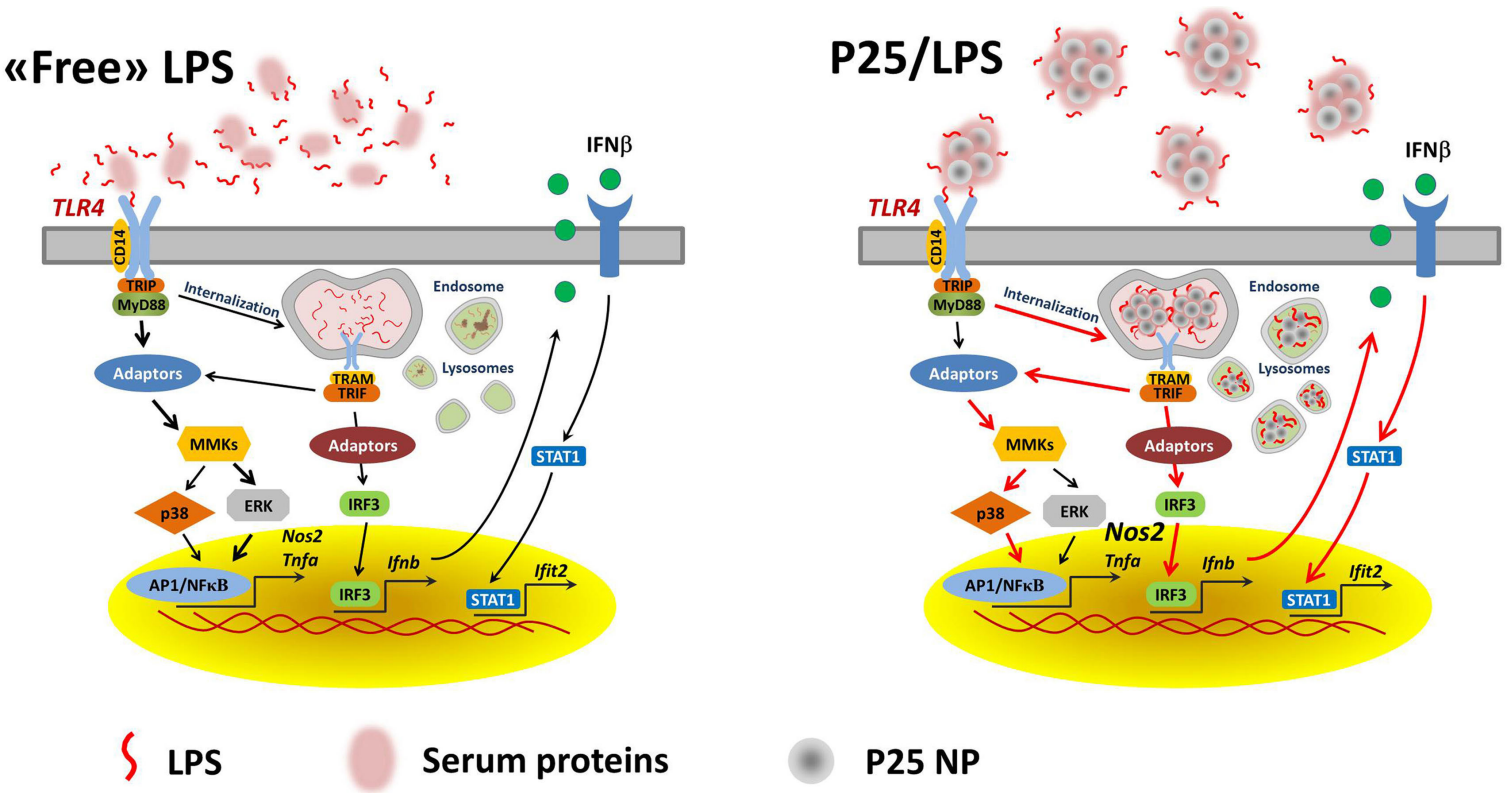
A model for the effects of adsorbed LPS in macrophages. Lines in red highlight the hypothesized prevalent and/or stimulated pathways in cells treated with P25/LPS.

We proposed a model in which mixing LPS with P25 leads to higher and more prolonged biological activity of the endotoxin and attributed the enhanced effects to the capability of triggering different transduction pathways and, in particular, of recruiting intracellular sites for signal transduction (20). As far as this issue is concerned, the results presented here substantially confirm that model. Indeed, the activation of p38 by P25/LPS, but not by free LPS, was sensitive to cytochalasin B and, consistently, cytochalasin B partially inhibited *Tnfa* induction by P25/LPS, but not by free LPS. On the other hand, the confocal images presented in Figure 7 indicate that the internalization of NP is much more evident for P25/LPS than for LPS-free NP, demonstrating that LPS adsorption enhances NP uptake, provides a facilitated access to endosomal sites of signal transduction and, likely, interferes with the processing of the LPS–TLR4 complex, thus ensuring a greater bio-persistence of the stimulus. However, cytochalasin B completely suppressed *Ifnb* and *Ifit2* induction by either free LPS or P25/LPS. This result is easily explained, if one considers that these genes are mainly TRIF-dependent and, hence, the relevant signals start from the endosomal compartment even in the case of free LPS, as demonstrated by the pivotal contribution by Kagan et al. (34) The evolution of the model on the basis of the data presented in this contribution is shown in Figure 9. In this model, we hypothesize that adsorbed LPS has enhanced and/or delayed effects due to an increased activity of internalization-dependent pathways.

The adsorption of LPS to TiO_2_ NP may account for the inflammatory changes observed *in vivo* after exposure to the nanomaterials under non-sterile conditions, such as those encountered in some working scenarios in which the exposure to TiO_2_ NP is associated with lung inflammation (35). On the other hand, unpublished results from our laboratory indicate that a substantial enhancement of biological effects upon interaction with NP is observed not only for LPS but also for other TLR agonists, such as polyI:C and zymosan. Thus, the acquisition by NP of a novel, more active biological identity after contact with biological fluids containing proteins and bioactive molecules may significantly enhance the inflammatory risks for individuals with conditions associated with increased levels of endogenous or exogenous TLR agonists.

In conclusion, these data suggest that, when included in the bio-corona of TiO_2_ NP, LPS exhibits enhanced and time-shifted pro-inflammatory effects. Thus, in assessing the hazard of NP in real life, the enhanced effects of adsorbed bioactive molecules should be taken into account.

## Author Contributions

MGB, MA, and MC performed the experiments; AC, MB, and SO provided reagents and performed the characterization of TiO_2_ NP; MGB and OB analyzed the data and wrote the manuscript; AC and EB critically read the manuscript. All the authors have approved the manuscript.

## Conflict of Interest Statement

The authors declare that the research was conducted in the absence of any commercial or financial relationships that could be construed as a potential conflict of interest.
